# Privacy-Preserving Federated Learning Framework for Multi-Source Electronic Health Records Prognosis Prediction

**DOI:** 10.3390/s25082374

**Published:** 2025-04-09

**Authors:** Huiya Zhao, Dehao Sui, Yasha Wang, Liantao Ma, Ling Wang

**Affiliations:** 1 Affiliated Xuzhou Municipal Hospital of Xuzhou Medical University, Xuzhou 221002, China; 2 National Engineering Research Center for Software Engineering, Peking University, Beijing 100871, China; zhaohuiya@stu.pku.edu.cn (H.Z.); dehaosui1@gmail.com (D.S.); wangyasha@pku.edu.cn (Y.W.); malt@pku.edu.cn (L.M.); 3 Key Laboratory of High Confidence Software Technologies, Ministry of Education, Beijing 100871, China

**Keywords:** federated learning, healthcare privacy, multi-institutional collaboration

## Abstract

Secure and privacy-preserving health status representation learning has become a critical challenge in clinical prediction systems. While deep learning models require substantial high-quality data for training, electronic health records are often restricted by strict privacy regulations and institutional policies, particularly during emerging health crises. Traditional approaches to data integration across medical institutions face significant privacy and security challenges, as healthcare providers cannot directly share patient data. This work presents MultiProg, a secure federated learning framework for clinical representation learning. Our approach enables multiple medical institutions to collaborate without exchanging raw patient data, maintaining data locality while improving model performance. The framework employs a multi-channel architecture where institutions share only the low-level feature extraction layers, protecting sensitive patient information. We introduce a feature calibration mechanism that ensures robust performance even with heterogeneous feature sets across different institutions. Through extensive experiments, we demonstrate that the framework successfully enables secure knowledge sharing across institutions without compromising sensitive patient data, achieving enhanced predictive capabilities compared to isolated institutional models. Compared to state-of-the-art methods, our approach achieves the best performance across multiple datasets with statistically significant improvements.

## 1. Introduction

Security and privacy concerns in AI-based clinical healthcare prediction using electronic health records (EHR) have become increasingly critical as healthcare institutions embrace data-driven approaches. While machine learning models have demonstrated remarkable capabilities in enhancing prognostic accuracy and resource optimization [1,2,3], the handling of sensitive healthcare data poses significant security challenges that must be addressed before widespread deployment.

Current approaches to EHR analysis typically involve processing sensitive patient information through feature embedding and sequential modeling [4]. These models extract private health information from multiple dynamic features (e.g., lab test values) into low-dimensional representations and learn temporal patterns from patient visits. However, this direct handling of sensitive medical data raises substantial privacy risks that must be carefully managed.

For practical deployment in medical scenarios, these AI systems must not only achieve clinical-grade accuracy [5] but also provide robust privacy guarantees. This dual requirement presents several security challenges in clinical applications:

- Feature Heterogeneity: Different healthcare institutions collect varying sets of clinical measurements and patient information, resulting in non-overlapping feature spaces. Traditional FL approaches assume shared feature spaces, leading to information loss or incompatibility when features differ across institutions.
- Task Diversity: Medical institutions often have different prediction targets based on their clinical focus (e.g., readmission prediction, mortality risk, length of stay), requiring models that can extract relevant features for diverse downstream tasks.
- Limited Knowledge Transfer: Existing approaches either sacrifice performance for privacy or fail to effectively transfer knowledge across heterogeneous data sources, especially when feature spaces only partially overlap.

To address these challenges, we correspondingly propose our solutions:

- We introduce a multi-channel embedding structure that accommodates both shared and institution-specific private features, allowing each institution to utilize its complete feature set without compromise.
- We propose a secure feature recalibration module that allows each institution to emphasize features most relevant to their specific prediction tasks, enabling personalized predictions while still leveraging shared knowledge.
- We implement shared low-level feature extraction layers that enable knowledge transfer across institutions while maintaining privacy, allowing institutions to benefit from collective insights without exposing sensitive information.

In this paper, we propose MultiProg, a privacy-preserving health representation learning framework, based on the multi-institutional federation that ensures end-to-end security of sensitive patient data. This approach enables several organizations to collaborate on the development of models without the need to directly share any local sensitive raw data among each other. In each round of training, collaborators (e.g., hospitals) are selected to train a model using local data. Only model updates are sent to the central server for aggregation to preserve data privacy. Concretely, a privacy-preserving multi-channel architecture is utilized to embed each clinical feature separately in the clinical representation learning to improve the compatibility across institutions with different feature sets while protecting feature relationships. Each collaborator can securely borrow useful feature extractors trained by other institutions even with different prediction targets, thus reducing the difficulty of its task-specific prediction layer. Finally, feature-wise recalibration is deployed to further adaptively emphasize critical features for various tasks through privacy-aware mechanisms. Our contributions are summarized as follows:

- We propose a secure federated clinical prediction framework, MultiProg, which builds privacy-preserving health representations by safely leveraging data from multiple institutions with various prediction tasks while maintaining strict data privacy. MultiProg helps to improve the performance of health evaluation (e.g., the prognosis of COVID-19 patients) effectively for all collaborators under data insufficiency scenarios, especially at the early stage of an emerging pandemic, without compromising sensitive patient information.
- We design a privacy-aware adaptive feature recalibration mechanism that securely adjusts the importance of different clinical features. This approach not only suppresses non-existent or less relevant features, but also enhances critical features for patients in diverse health conditions. Through shared feature extraction channels, the framework enables embedding of patients with different recorded features into a unified clinical feature space, while protecting each institution’s data characteristics and confidentiality.
- We validate our framework’s effectiveness through secure collaboration between multiple medical institutions (i.e., Tongji Hospital in China, HM Hospitals in Spain, and a private nephrology center) on various prediction tasks (i.e., length of stay prediction for COVID-19 patients, and mortality risk prediction for chronic kidney disease patients). Experimental results demonstrate improved prediction performance across all participating institutions while maintaining data privacy. The source code is available at GitHub https://github.com/anonymous20250128/MultiProg (accessed on 28 January 2025).

## 2. Related Work

In this section, we review the relevant literature that provides context for our research. First, we explore the clinical background to establish the medical foundation and challenges in the specific healthcare domain we address. Next, we examine various solutions for the data scarcity problem, which remains a significant barrier to effective AI applications in healthcare. Finally, we investigate federated learning for healthcare, a promising approach that enables collaborative model training while preserving data privacy and addressing regulatory concerns in medical settings.

### 2.1. Clinical Background

Healthcare systems worldwide face persistent challenges from disease outbreaks and public health emergencies, which continue to test the resilience and adaptability of medical infrastructure. Emergency health crises impose extraordinary demands on public health systems and community service providers [6], often exceeding available resources and operational capacities. The global medical resource shortages experienced during the COVID-19 pandemic serve as a compelling example. When healthcare resources become constrained, the resulting delays in treatment can lead to deteriorating patient outcomes [7], creating a cascading effect that exacerbates the overall impact of the health crisis.

With the development of healthcare together with the update of storage, valuable digital information stored in electronic health records (EHR) has opened new opportunities for researchers to make secondary use of these records for various clinical applications [8,9,10]. Many deep learning-based models have been developed to mine the massive EHR data due to the remarkable representation learning ability of neural networks. These methods have shown superior performance in many tasks, including mortality prediction [11,12,13], patients subtyping [14], and diagnosis prediction [14,15,16,17,18,19]. Though the medical tasks vary from each other, extracting advanced clinical features and learning the compressed representation of the sparse EHR data are fundamental procedures of clinical healthcare prediction. Such representations can characterize patients’ information in low-dimensional space, thus making the mortality risk and disease diagnosis prediction easier.

However, training deep learning-based models usually needs a large amount of data with high diversity that represents the practical application environment. The quantity of labeled data is much less for some rare diseases, which cannot support a model to be trained thoroughly.

### 2.2. Solutions for the Data Scarcity Problem

To address the challenge of limited data availability, researchers have explored various approaches to enhance model performance.

Choi et al. [20] leverages the inherent multilevel structure (e.g., the relationship between diagnosis codes and treatment codes) of EHR data to improve learning efficiency. Ma et al. [21] introduce external well-organized ontology information (e.g., International Classification of Diseases Codes) to represent the medical concept as a combination of its ancestors in the ontology via an attention mechanism. However, such relationships and ontology information are often not easy to access in clinical practice. In addition, ontological information is usually designed to handle the medical codes. Thus, it is not suitable for dealing with numerical lab tests, which also are essential clinical features for capturing health status. For example, there is no kind of normal structured information of relationship information among lab test values (e.g., blood glucose, hemoglobin).

On the other hand, some researchers try to explore the existing EHR data. Gupta et al. [22] trains a deep RNN to identify several patient phenotypes on time series from the MIMIC-III dataset and then uses the features extracted by the RNN to build classifiers for identifying previously unseen phenotypes. However, these methods can only be utilized for the same clinical feature sets between source and target datasets. TimeNet is pre-trained on non-medical time series in an unsupervised manner and further utilized to extract features for clinical prediction [23]. Nevertheless, the trained parameters for the non-medical data may not be suitable for the specific clinical task, leading to negative transfer and limited performance.

### 2.3. Federated Learning for Healthcare

While traditional approaches focus on extracting more information from limited data, federated learning presents a fundamentally different solution by enabling collaborative learning across institutions while preserving data privacy. This paradigm has been extensively studied in financial security, allowing model learning across multiple institutions without direct data sharing. In this approach, collaborators train a shared global model with a server across multiple decentralized clients holding local data samples, and then only the updated results are aggregated to the server [24]. After joint optimization, the server returns the global state to clients, and continues to accept the updated data calculated by each client in the new global state.

In the medical record analysis area, for the protection and respect of patients’ privacy, the hospital’s specific medical-related data did not allow leakage and sharing without permission [25]. Sheller [26,27] introduces the first use of federated learning to perform brain tumor segmentation. Murphy et al. [28] proposes a better representation of patient, in each stage the model is trained with one dataset federated. Although Huang et al. [29] claimed that patient clustering improves efficiency, in real scenarios we cannot obtain distribution of data and create clusters. These early applications of federated learning in healthcare, while promising, are still limited by challenges in handling heterogeneous data structures across institutions and the need for more efficient communication protocols.

## 3. Problem Formulation

In this section, we formally define the collaborative medical modeling problem, establishing the deep learning framework for multiple institutions to jointly develop a foundation model while addressing data heterogeneity and diverse clinical objectives.

Medical institutions aim to build a foundation model for robust representation learning and accurate task predictions. Consider *N* medical collaborators (e.g., hospitals) $C^{1},\cdots ,C^{N}$ with their respective data $D^{1},\cdots ,D^{N}$ and clinical prediction targets $Y^{1},\cdots ,Y^{N}$.

For instance, in COVID-19 departments, many hospitalized patients require intensive care monitoring. These departments need to predict patients’ remaining time in ICU (i.e., length of stay) to assess illness severity and optimize medical resource allocation [30]. In contrast, nephrology departments may focus on predicting mortality risk for patients with end-stage renal disease (ESRD). Specifically, for a given collaborator $C^{n}$:

(1) $$D^{n}=\{\left(r_{i}^{n},y_{i}^{n}\right){\}}_{i=1}^{M^{n}},$$

where $r_{i}^{n}$ is the medical records of patient *i*, $M^{n}$ is the data size of collaborator $C^{n}$, and $y_{i}^{n}$ represents the target label of patient *i*. To be more exact, hospitals usually record various medical feature sets (e.g., medical biomarkers, vital signs) that change over time during the patient’s stay. Therefore, we can model the medical records $r_{i}^{n}$ as a sequence of feature vectors:

(2) $$r_{i}^{n}=\left(\begin{array}{ccc} r_{1,1} & \cdots & r_{1,T}\\ \vdots & \ddots & \vdots \\ r_{x^{n},1} & \cdots & r_{x^{n},T} \end{array}\right).$$

The feature sets shared by collaborator *i* and collaborator *j* can be demonstrated as

(3) $$X^{i}\cap X^{j}\neq ⌀,\;\;\;\forall D^{i},D^{j},\;i\neq j$$

where $X^{n}$ is the feature sets recorded in collaborator $C^{n}$. A conventional aggregating training method is to put all institutions’ data with the same feature sets and the same prediction targets together to train a model. However, it is tough to satisfy this condition, especially at the early stage of an emerging epidemic. Furthermore, it will also cause unavoidable privacy leakage. Federated learning decentralizes deep learning by removing the need to pool data into a single location. Instead, the model is trained in multiple iterations at different sites. For example, say two COVID-19 institutions and one cardiology institution decide to team up, jointly building models to predict the length-of-stay of COVID-19 patients and perform the sepsis early detection of other patients. Concretely, in this paper, multitasking multi-institutional federation learning is proposed to improve the performance of the various prediction tasks for all collaborators with different feature sets.

(4) $$r^{n}\cap r^{j}=⌀,\;\;\;X^{n}\neq X^{j},\;\;\;Y^{n}\neq Y^{j},\;\;\;\forall D^{n},D^{j},\;n\neq j$$

It deals with the problem of exceeding the scope of basic federated learning, where for any two institutions *n* and *j*: their record sets have no intersection ($r^{n}\cap r^{j}=⌀$), their feature spaces differ ($X^{n}\neq X^{j}$), and they focus on different prediction tasks ($Y^{n}\neq Y^{j}$).

## 4. Methodology

**Figure 1:**
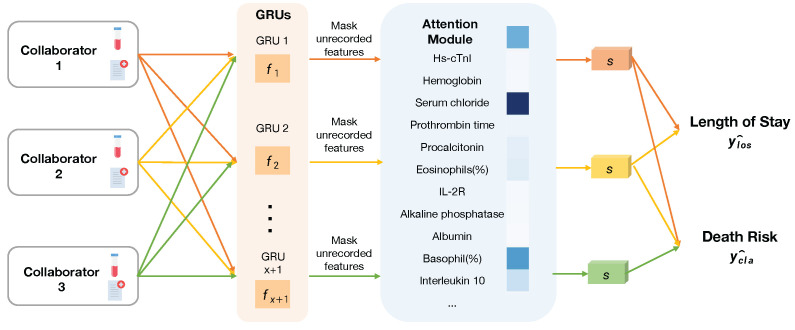
Overall architecture of our proposed MultiProg framework.

Our proposed framework, MultiProg, is a secure clinical representation learning approach designed to handle heterogeneous sensitive clinical data from different institutions while preserving privacy. The framework consists of three main components: (1) a multi-channel embedding structure that processes different feature sequences independently under secure protocols, whose design allows each institution to effectively process their unique data while maintaining a shared learning framework, addressing the data heterogeneity challenge prevalent in healthcare institutions; (2) shared low-level feature extraction layers that enable privacy-compliant cross-institutional knowledge transfer without exposing raw data: by sharing only model parameters of these extraction layers rather than actual patient data, our approach maintains regulatory compliance while still facilitating valuable knowledge transfer between institutions; and (3) a secure feature calibration module that addresses missing or irrelevant features specific to each institution. This architecture allows for robust representation learning while protecting institutional data variations and sensitive patient information. The overall structure of our secure framework is illustrated in Figure 1.

### 4.1. Sequential Medical Records Representation

In order to facilitate each hospital with different characteristics as participants to make better use of the collaborative learning framework, we utilize the multi-channel clinical sequence embedding [31] for each individual patient, with each channel responsible for processing specific types of sequential medical records.

Through these channels, clinical time series data are embedded into a unified feature space. For datasets containing common features, the corresponding GRU-based feature extractors are jointly trained, allowing them to capture more robust and generalizable patterns from the shared feature space. Specifically, each feature is embedded by RNN separately:

(5) $$f_{x}={\mathrm{GRU}}_{x}\left(r_{x,1},...,r_{x,T}\right)$$

Furthermore, the demographic baseline data (e.g., age, gender, primary disease) $base_{1},base_{2},...,base_{b}$ are embedded into the same hidden space of $f_{x}$ as hidden size *h*, where $W^{base}\in R^{m\times h}$ is a learnable embedding matrix.

(6) $$f_{x+1}=W^{base}\cdot base$$

Thus, all the data of the patient can be represented by a matrix $F={\left(f_{1},\cdots ,f_{x},f_{x+1}\right)}^{⊤}$, which is a sequence of vectors, and each vector represents one feature of the patient over time.

### 4.2. Multi-Institutional Federated Learning with Various Feature Sets

For the federated learning of EMR analysis, a centralized server maintains the global deep neural network and each participating hospital receives a copy to train on their local dataset. To preserve the privacy of health data, each client trains the local model using their local dataset in each round of training, then encrypts the updated parameters and uploads them to the server. Mathematically, if we denote *N* participating collaborators with their respective datasets, in each communication round *t*, the server aggregates local model updates using FedAvg:

(7) $$w^{t+1}=\sum_{n=1}^{N}\frac{M^{n}}{M}w_{n}^{t+1}$$

where $M^{n}$ is the number of samples at collaborator $C^{n}$, and *M* is the total number of samples across all hospitals.

Our multi-channel embedding operates within this FL framework specifically to handle heterogeneous feature sets across hospitals. For each hospital, we separate features into shared features (common across hospitals) and private features (unique to that hospital). The multi-channel embedding can be represented as

(8) $$F_{n}=\left[F_{n}^{s},F_{n}^{p}\right]$$

where $F_{n}^{s}$ and $F_{n}^{p}$ represent embeddings from shared and private GRU channels, respectively. During federated training, only the parameters of shared GRU channels are updated collaboratively, whereas private GRU channels are updated locally.

During data preprocessing, to keep feature value consistency, the data undergo unified standardization operations. For common features, collaborators share the metadata used for standardization (e.g., mean value and standard deviation of the shared features) without exchanging raw data. All collaborators embed their sequential records via the corresponding GRU channels in the federated framework. Hospitals with shared features borrow useful information from each other by jointly training the feature extractors in common.

The prediction tasks usually differ among collaborators, and thus the final prediction layer is supposed to be private for some hospitals. Even so, they still expect to share the low-level layers with other collaborators to jointly obtain robust embedding. The feature recalibration mechanism helps each hospital focus on features most relevant to its specific task through an attention mechanism, allowing effective knowledge sharing even when prediction tasks differ.

### 4.3. Multi-Channel Feature Recalibration

In order to avoid the distraction of unrecorded or useless features for each collaborator, feature recalibration is designed to automatically suppress the non-existent features for each hospital, and at the same time adaptively enhance important features for patients in diverse health conditions.

Based on shared feature extraction channels, all collaborators can embed patients with different recorded features in the same clinical feature space. This unified representation enables physicians to perform comprehensive cohort studies and patient group analyses. The feature recalibration mechanism guarantees the individuation of each collaborator. As a result, such a federated representation learning framework can jointly improve the prediction performance for each collaborator and provide reasonable interpretability.

Specifically for collaborator $C^{n}$, we calculate the queries, keys, and values for *F* obtained in the multi-variable sequence representation learning layer:

(9) $$q_{i}=W_{i}^{q}\cdot \bar{f},$$

(10) $$k_{i}=W_{i}^{k}\cdot f_{i},$$

(11) $$v_{i}=W_{i}^{v}\cdot f_{i},$$

where $W^{q}$, $W^{k}$, and $W^{v}$ are the learnable projection matrix, respectively, and *i* is from 1 to n+1. The attention weights are calculated as

(12) $$\alpha_{1},...,\alpha_{x},\alpha_{x+1}=\mathrm{Softmax}\left(\zeta_{1},...,\zeta_{x},\zeta_{x+1}\right),$$

where

$$\zeta_{i}=\;\left\{\begin{array}{cc} q_{i}\cdot k_{i}, & \mathrm{if}\;r_{i}\;\mathrm{recorded}\;\mathrm{in}\;C^{n}\\ mask, & \mathrm{if}\;r_{i}\;\mathrm{unrecorded}\;\mathrm{in}\;C^{n} \end{array}\right.$$

where mask is a negative number with large absolute value. The health status representation *s* can be obtained as

(13) $$s=\sum_{i=1}^{N}\alpha_{i}\cdot v_{i}.$$

### 4.4. Prediction Layer

Collaborators build their own prediction layer based on the jointly trained embedding module. The classification task (e.g., mortality prediction, sepsis prediction) can be computed as

(14) $${\hat{y}}_{cla}=\sigma \left(W_{cla}\cdot s+b_{cla}\right),$$

where $W^{cla}$ and $b^{cla}$ are the learnable matrix and bias term, respectively. Assuming $M^{n}$ is the total number of samples of collaborator $C^{n}$, the final loss can be denoted as binary cross-entropy loss:

(15) $$L_{cla}=-\frac{1}{S}\sum_{i=1}^{S}\left[y_{cla}^{i}\log\left({\hat{y}}_{cla}^{i}\right)+\left(1-y_{cla}^{i}\right)\log\left(1-{\hat{y}}_{cla}^{i}\right)\right].$$

For the regression task, such as length-of-stay prediction, which aims to predict the remaining days to outcome at each record of patients, the calculation is

(16) $${\hat{y}}_{reg}=W^{reg}\cdot s+b^{reg},$$

Similarly, $W_{reg}$ and $b_{reg}$ are learnable. The final loss can be regarded as mean squared error (MSE):

(17) $$L_{reg}=-\frac{1}{S}\sum_{i=1}^{S}{\left(y_{reg}^{i}-{\hat{y}}_{reg}^{i}\right)}^{2}.$$

Finally, hospitals with different tasks can choose to perform fine tuning separately at the local level. The process of multitask federated modeling is presented in Algorithm 1.
**Algorithm 1** Multitask collaborative training methodinitialize $w_{0}$**while** not convergence **do**   $G_{t}\leftarrow 0$   **for** each Collaborator $C_{n}$ **do**     freeze and mask unused parameters in $w_{t-1}$     $G_{t}\leftarrow G_{t}+▽l\left(w_{t-1};b_{t}\right)$   **end for**   $W_{t}\leftarrow Adam\left(W_{t-1},G_{t}\right)$**end while**

In Algorithm 1, $G_{t}$ represents the accumulated gradient across all collaborators at iteration *t*, and $l(w_{t-1};b_{t})$ denotes the loss function evaluated on the model parameters $w_{t-1}$ using the mini-batch $b_{t}$. We use the Adam optimizer for its effectiveness with sparse gradients [32].

## 5. Experiments

In this section, we present a comprehensive evaluation of our proposed approach. We first introduce the datasets used for experimentation, followed by our experimental setup, including evaluation metrics and baseline methods for comparison. We then analyze the results from both quantitative and qualitative perspectives to demonstrate the effectiveness of our method.

We adopt three real-world datasets: TJH, CDSL, and ESRD datasets to perform mortality prediction and length of stay (LOS) prediction tasks. The performance is demonstrated via the case study of a patient’s dynamic health trajectory. The source code of MultiProg and the interaction system are available at the GitHub repository (https://github.com/anonymous20250128/MultiProg) (accessed on 28 January 2025).

### 5.1. Medical Institution Collaborators

- TJH [1]: comprises anonymized EHR data from 485 COVID-19 patients admitted to Tongji Hospital, China, between 10 January and 24 February 2020. The dataset includes 74 lab tests and vital signs, all of which are numerical features, as well as two demographic features (age and gender).
- CDSL [33]: This dataset is derived from the HM Hospitales EHR system in Spain and consists of anonymized records of 4479 patients admitted with a confirmed or suspected diagnosis of COVID-19. CDSL offers a rich variety of medical features, including comprehensive details on diagnoses, treatments, admissions, ICU stays, diagnostic imaging tests, laboratory results, and patient discharge or death status.
- ESRD [34]: The end-stage renal disease (ESRD) dataset comprises data from 656 peritoneal dialysis patients, including 13,091 visit records collected over a 12-year period, from 1 January 2006 to 1 January 2018. This longitudinal dataset features patients’ baseline information, visit records, and clinical outcomes, offering a unique perspective on long-term peritoneal dialysis treatment and patient progression.

**Figure 2:**
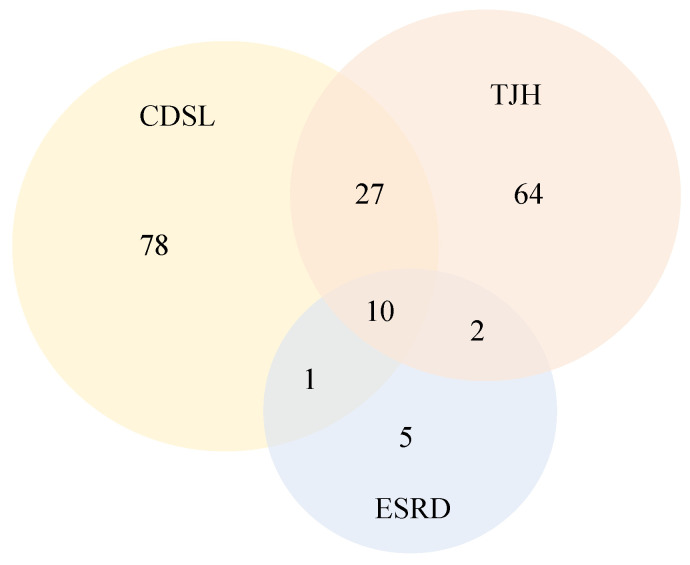
Intersection relationship of clinical features recorded by collaborators.

The intersection relationship of clinical features recorded in these collaborators is intuitively shown in Table 1 and Figure 2. Numbers denoted in each part represent the number of features. For more information about dataset TJH and CDSL, you can refer to Appendix A.

### 5.2. Experimental Setup

#### 5.2.1. Tasks and Evaluation Metrics

We perform mortality prediction tasks on all three datasets and LOS prediction tasks on the TJH and CDSL datasets. The mortality predication task can be formulated as a binary classification problem and labeled by $y_{hat}\in \left\{0,1\right\}$, signifying whether the patient will succumb by the end of the ICU stay. The LOS prediction can be regarded as a regression problem, and we take the remaining days *t* in ICU as the ground truth LOS label.

We assess the binary classification performance using AUROC and AUPRC. AUROC (area under the receiver operating characteristic curve) measures classification performance across thresholds. AUPRC (area under the precision–recall curve) evaluates precision–recall trade-offs in imbalanced datasets. Here, we emphasize AUPRC as the main metric due to it being informative when dealing with highly imbalanced and skewed datasets [35,36], as shown in our selected datasets.

For regression tasks, three primary metrics are commonly employed to evaluate prediction performance: MSE, MAE, and RMSE. Here, we emphasize MSE as the main metric.

#### 5.2.2. Baseline Approaches

We introduce several deep learning-based models as our baseline approaches without additional labeled data or external ontology resources.

- RNN [37] is the most popular framework to learn the abstract embedding of variable-length time series.
- GRU [38] is the basic gated recurrent unit network.
- LSTM [39] is a variant of the recurrent neural network, capable of learning long-term dependencies.
- RETAIN [16] is the deep-based reverse time attention model for analyzing EHR data. It utilizes a two-level neural attention module to attend important clinical visits and features.
- M3Care [40] is an end-to-end model compensating the missing information of the patients with missing modalities to perform clinical analysis.
- AICare [34] consists of a multi-channel feature extraction module and an adaptive feature importance recalibration module to build the health status embedding for each patient individually.

For our baseline methods, we employed a consistent hyperparameter configuration with hidden dimension size of 32, dropout rate of 0.1, and GELU activation function as the default choice. For the RNN, GRU, and LSTM models, we configured a single-layer architecture without bidirectional encoding (bidirectional = False). Regarding RETAIN, M3Care, and AICare implementations, we primarily followed the original implementations available in the code repositories referenced in their respective papers.

#### 5.2.3. Implementation Details

All runs are trained on a single Nvidia RTX 3090 GPU with CUDA 11.8. The server’s system memory (RAM) size is 64 GB. We implement the model in Python 3.11.4, PyTorch 2.0.1 [41], PyTorch Lightning 2.0.5 [42], and pyehr [43,44].

AdamW [45] is employed with a batch size of 1024 patients. All models are trained for 50 epochs with an early stopping strategy based on AUPRC after 10 epochs without improvement. The learning rate 0.01,0.001,0.0001 and hidden dimensions 64,128 are tuned using a grid search strategy on the validation set. The searched hyperparameter for MultiProg is 32 hidden dimensions and 0.001 learning rate. Performance is reported in the form of mean ± std with applying bootstrapping on all test set samples 10 times for all three datasets [34].

### 5.3. Quantitative Analysis

As demonstrated in Table 2 and Table 3, our proposed method MultiProg consistently outperforms all baseline approaches across different evaluation metrics, indicating its superior capability in learning robust representations.

For mortality prediction, MultiProg achieves significant improvements for both datasets. On the CDSL dataset, it attains an AUPRC of 87.45% and an AUROC of 97.89%, substantially surpassing all comparative baselines. The performance gains are even more pronounced on the TJH dataset, where MultiProg reaches an AUPRC of 99.70% and an AUROC of 99.78%. In terms of LOS prediction, MultiProg reduces the MSE to 33.02 and 3.91 on the TJH and CDSL datasets, respectively.

In our statistical analysis, we conducted hypothesis testing, with the null hypothesis (H0) that the MultiProg method shows no significant performance difference compared to other methods (such as AICare, RETAIN, etc.), and the alternative hypothesis (H1) that MultiProg significantly outperforms other methods. The results demonstrate that our method achieves statistically significant advantages across nearly all datasets and metrics when compared to the best baseline methods. Specifically, for both TJH and CDSL datasets, *p*-values are below 0.001 across all metrics, indicating strong statistical significance. Even for the more challenging ESRD dataset, *p*-values remain below 0.05, still demonstrating significant performance advantages. These rigorous statistical tests provide strong evidence that the superiority of our method is not due to random chance. The results are displayed in Table 4.

Our experimental results clearly demonstrate that models trained with a larger number of collaborating institutions achieve superior performance compared to those trained with fewer participants. This finding suggests that MultiProg could enable smaller healthcare facilities and rural hospitals to access high-quality AI algorithms that benefit from collective expertise and diverse training data.

## 6. Discussion

- Limitations and future directions in privacy protection: Our approach builds upon a traditional federated learning framework, inheriting its fundamental privacy guarantees such as secure aggregation and differential privacy. However, when dealing with heterogeneous data, specific privacy challenges remain. In particular, when data distributions across different institutions exhibit significant variations, models may become more vulnerable to membership inference attacks even with differential privacy applied [46]. In the future, we plan to explore hybrid schemes combining local differential privacy with hierarchical encryption [47], tailoring privacy protection levels for scenarios with extreme data heterogeneity.
- Missing data handling: We handle missing data using standard mean imputation for continuous features and mode imputation for categorical features. More sophisticated approaches could further improve model performance when dealing with sparse or incomplete patient records. For example, incorporating uncertainty estimation for imputed values could be integrated into our feature recalibration module, as demonstrated by Nazabal et al. in their probabilistic approach to missing data [48].
- Extended ethical and regulatory considerations: While our method complies with existing ethical standards and regulations (such as HIPAA and GDPR), regulatory gray areas may emerge in multi-institutional collaborative settings. Particularly when international institutions collaborate, differences in data protection regulations across countries may lead to compliance challenges. Additionally, our current framework lacks ethical considerations for specific disease categories (such as rare diseases), where patient identities might be exposed even with anonymized data.

## 7. Conclusions

In this work, we propose a secure federated learning model MultiProg, which enables privacy-preserving collaboration among hospitals, healthcare institutions, and research centers to build a model that benefits all participants while ensuring data security. MultiProg allows every participant to keep control of its own clinical data without needing to directly share any local sensitive raw data. The framework leverages a privacy-aware multi-channel architecture that securely embeds each clinical feature separately in clinical representation learning, enabling secure cross-institutional collaboration despite different feature sets. The experiments with real-world collaborators show that MultiProg can improve the performance of health evaluation effectively for all collaborators. This performance improvement is especially useful under data insufficiency settings, especially at the early stage of an emerging pandemic. 

## Figures and Tables

**Table 1:** Shared or private features recorded by collaborators.

TJH	CDSL	ESRD
All		
albumin	ALBUMINA	Albumin
Urea	UREA	Urea
hemoglobin	Hemoglobina	Hemoglobin
...	...	...
TJH-CDSL		
HCO3-	HCO3	-
Eosinophil	Eosinfilos	-
...	...	
TJH-ESRD		
WBC	-	WBC
Potassium	-	Potassium
...		...
CDSL-ESRD		
-	CLORO	Cl
	...	...

**Table 2:** Prediction performance of mortality prediction task on TJH, CDSL, and ESRD datasets. MultiProg-2 collaborates two datasets: TJH and CDSL. MultiProg-2 collborates all three datasets. **Bold** indicates the best performance. Performance is reported in the form of mean ± std with bootstrapping applied on all test set samples 10 times for all three datasets. All metric scores are multiplied by 100 for readability purposes.

Methods	TJH		CDSL		ESRD	
	AUPRC (↑)	AUROC (↑)	AUPRC (↑)	AUROC (↑)	AUPRC (↑)	AUROC (↑)
RNN	98.08 ± 1.46	98.52 ± 1.16	50.83 ± 4.07	79.56 ± 2.08	40.17 ± 4.63	49.09 ± 4.48
GRU	98.12 ± 1.70	98.34 ± 1.41	80.21 ± 4.01	95.87 ± 0.95	42.70 ± 5.20	50.63 ± 4.47
LSTM	98.62 ± 1.54	99.01 ± 1.20	64.60 ± 4.86	89.94 ± 1.55	65.07 ± 7.00	77.72 ± 3.88
RETAIN	98.78 ± 1.18	99.13 ± 0.81	75.88 ± 4.12	93.66 ± 1.17	65.24 ± 6.08	75.32 ± 3.84
AICare	99.14 ± 0.82	99.11 ± 0.82	83.42 ± 3.71	95.78 ± 1.00	69.11 ± 6.08	76.30 ± 4.08
M3Care	97.20 ± 2.63	98.36 ± 1.57	71.63 ± 4.69	92.22 ± 1.49	**70.42 ± 6.01**	75.75 ± 4.27
MultiProg-2	99.68 ± 1.68	**99.83 ± 1.34**	84.88 ± 2.82	96.91 ± 0.95	-	-
MultiProg	**99.70 ± 1.59**	99.78 ± 1.38	**87.45 ± 4.50**	**97.89 ± 1.03**	61.84 ± 6.77	**78.34 ± 4.13**

**Table 3:** Prediction performance of LOS prediction task on TJH and CDSL datasets. **Bold** indicates the best performance. Performance is reported in the form of mean ± std with bootstrapping applied on all test set samples 10 times for all three datasets.

Methods	TJH			CDSL		
	MSE (↓)	RMSE (↓)	MAE (↓)	MSE (↓)	RMSE (↓)	MAE (↓)
RNN	38.40 ± 16.30	6.05 ± 1.34	3.74 ± 0.77	5.64 ± 2.07	2.33 ± 0.44	0.63 ± 0.07
GRU	33.51 ± 17.14	**5.58 ± 1.53**	3.15 ± 0.78	5.64 ± 2.08	2.33 ± 0.44	0.58 ± 0.07
LSTM	38.20 ± 18.70	5.97 ± 1.59	3.05 ± 0.84	5.70 ± 2.10	2.35 ± 0.44	**0.49 ± 0.07**
RETAIN	44.21 ± 20.73	6.46 ± 1.58	3.79 ± 0.88	5.83 ± 2.01	2.38 ± 0.42	0.82 ± 0.07
AICare	38.87 ± 20.40	5.99 ± 1.73	**2.95 ± 0.86**	5.49 ± 2.06	2.30 ± 0.44	0.55 ± 0.07
M3Care	34.29 ± 16.92	5.66 ± 1.49	3.17 ± 0.79	5.69 ± 2.07	2.34 ± 0.44	0.59 ± 0.07
MultiProg	**33.02 ± 9.68**	5.69 ± 0.82	4.38 ± 0.60	**3.91 ± 0.97**	**1.96 ± 0.26**	0.75 ± 0.05

**Table 4:** Statistical comparison of MultiProg versus best competing methods across all datasets and metrics. **Bold** indicates the best performance. Significance levels are denoted as: *** (*p* < 0.001), * (*p* < 0.05).

Dataset	MultiProg Performance		Best Competitor		Statistical Comparison	
	AUPRC(↑)	AUROC(↑)	AUPRC	AUROC	*p*-Value	Significance
TJH	**99.70 ± 1.59**	**99.78 ± 1.38**	AICare (99.14 ± 0.82)	RETAIN (99.13 ± 0.81)	$8.71\times 10^{-8}/8.93\times 10^{-12}$	***/***
CDSL	**87.45 ± 4.50**	**97.89 ± 1.03**	AICare (83.42 ± 3.71)	GRU (95.87 ± 0.95)	0.00/0.00	***/***
ESRD	61.84 ± 6.77	**78.34 ± 4.13**	**M3Care (70.42 ± 6.01)**	LSTM (77.72 ± 3.88)	$3.33\times 10^{-63}/1.67\times 10^{-2}$	***/*

## Data Availability

TJH and CDSL datasets used in this study are publicly available. It is described in detail at https://www.cell.com/patterns/fulltext/S2666-3899(24)00050-3, (accessed on 28 January 2025) and can be accessed through Appendix A. The private dataset ESRD is available upon reasonable request to the authors, subject to ethical and privacy considerations. Researchers interested in accessing the private dataset should contact the corresponding author and follow the guidelines described in https://www.cell.com/patterns/fulltext/S2666-3899(23)00294-5?__cf_chl_tk=k_ekcE10bJAaGqBfl0ImGYYuyNX3EgRG.oy4DN4DWa4-1730540756-1.0.1.1-Gtrg4nES_CsGX.xAY6NErcagFXwgMsoH1eLyBeWB73A, (accessed on 28 January 2025).

## Appendix A. Online Resources

- The Tongji Hospital COVID-19 Collaborator is introduced at https://www.nature.com/articles/s42256-020-0180-7 (accessed on 24 October 2023).
- The HM Hospitals COVID-19 Collaborator is introduced at https://www.hmhospitales.com/prensa/notas-de-prensa/comunicado-covid-data-save-lives (accessed on 15 October 2023).
